# Psychopathological Features of Bipolar Depression: Italian Validation of the Bipolar Depression Rating Scale (I-BDRS)

**DOI:** 10.3389/fpsyg.2018.01047

**Published:** 2018-06-21

**Authors:** Angelo Bruschi, Marianna Mazza, Giovanni Camardese, Salvatore Calò, Claudia Palumbo, Laura Mandelli, Antonino Callea, Alessio Gori, Marco Di Nicola, Giuseppe Marano, Michael Berk, Guido di Sciascio, Luigi Janiri

**Affiliations:** ^1^Institute of Psychiatry and Psychology, Department of Geriatrics, Neuroscience and Orthopedics, Catholic University of Sacred Heart, Rome, Italy; ^2^Istituto di Psicopatologia, Rome, Italy; ^3^Department of Mental Health, ASL Viterbo, Rome, Italy; ^4^Department of Psychiatry, Policlinico Hospital Bari, Bari, Italy; ^5^Department of Mental Health, ASL Lecce, Lecce, Italy; ^6^Esine Hospital, ASST Valcamonica, Esine, Italy; ^7^Department of Biomedical and Neuromotor Sciences, University of Bologna, Bologna, Italy; ^8^Department of Social Science, LUMSA University, Rome, Italy; ^9^Department of Human Science, LUMSA University, Rome, Italy; ^10^Department of Education and Psychology, University of Florence, Florence, Italy; ^11^IMPACT Strategic Research Centre, School of Medicine, Deakin University, Geelong, VIC, Australia; ^12^Orygen Youth Health Research Centre, Florey Institute for Neuroscience and Mental Health and the Department of Psychiatry, University of Melbourne, Melbourne, VIC, Australia

**Keywords:** bipolar disorder, bipolar depression rating scale, validation, unipolar, depression, mixed state, mania

## Abstract

**Background:** Aim of the study was the validation of the Bipolar Disorder Rating Scale (BDRS) in an Italian population. Secondary aim was the evaluation of differences between unipolar and bipolar depression and between bipolar I and II depressed patients.

**Method:** 125 Bipolar Disorder and 60 Major Depressive Disorder patients were administered an Italian translation of the BDRS (I-BDRS), Hamilton Depression Rating Scale (HDRS), Montgomery-Asberg Depression Rating Scale (MADRS), Young Mania Rating Scale (YMRS) and Temperament and Character Inventory-Revised (TCI-R).

**Results:** I-BDRS showed considerable validity and reliability. Factor analysis found 3 subscales, two linked to depressive symptoms and one to mixed symptoms. Measures concerning depression (MADRS and HAM-D) were positively related to the I-BDRS's subscales, but mostly to the two subscales measuring depression. In mixed symptoms, the mean of the bipolar group was significantly higher than the unipolar group suggesting that the BDRS was able to distinguish between unipolar and bipolar depressed patients.

**Conclusion:** I-BDRS is a valid scale for the measurement of depression in BD patients, with a notable internal consistency (Cronbach's α 0.82), a significant consistency between items/total (Cronbach's α from 0.80 to 0.82) and positive correlation with other scales (MADRS *r* = 0.67, *p* < 0.001; HDRS *r* = 0.81, *p* < 0.001; YMRS *r* = 0.46 *p* < 0.0001). The mixed state sub-scale shows usefulness in differentiating bipolar from unipolar patients. I-BDRS could be a sensitive tool, both in pure depression and in mixed states, and could be used in the everyday screening and treatment of Bipolar Disorder.

## Introduction

Although mania or hypomania are considered the pathognomonic characteristics of bipolar disorder (BD), depression is more common than manic symptoms during lifetime of bipolar patients (Judd et al., 2002). Hence, psychometric instruments for the assessment of bipolar depressive symptoms have a capital role in both clinical research and everyday psychiatric practice. Apart from Bipolar Depression Rating Scale (Berk et al., 2007), other scales have been largely developed and validated on unipolar depressed patients and lack of sensitivity and accuracy to discriminate different psychopathological nuances of bipolar disorder. Bipolar depression, indeed, has its own unique and distinct clinical profile that differs from unipolar depression. Bipolar patients not only spend most of their time suffering from syndromal or sub-syndromal depression (Judd et al., 2002), but their depressive symptoms are even greater in bipolar II disorder (Judd et al., 2003). The lifetime prevalence of bipolar spectrum disorder is 4.5% (instead of 16.2% of major depressive disorder, MDD) (Kessler et al., 2003; Merikangas et al., 2011), with an equal gender distribution, except for bipolar II disorder that is more common in females (Nivoli et al., 2011). Age of onset is earlier (average 6 years before compared to major depression) with episodes of illness that tend to be shorter and highly relapsing, abrupt beginning and sudden end (Weissman et al., 1996). Rate of suicidality is higher, with a lifetime prevalence of 17% in bipolar I disorder and 24% in bipolar II disorder, compared to 12% in unipolar depression (Rihmer and Kiss, 2002; Kessler et al., 2005). With regard to clinical presentation, individuals with bipolar disorder are more likely to report atypical depressive features, psychosomatic reactions and mood reactivity (Angst and Sellaro, 2000; Goodwin and Jamison, 2007), with symptoms such as hypersomnia, hyperphagia and pressured speech (Cuellar et al., 2005). Psychotic aspects and substance abuse are frequent especially in young people (Tohen et al., 1998; Mitchell et al., 2001; Joslyn et al., 2016).

Bipolar depression is often entangled by the presence of sub-threshold manic symptoms (so-called “mixed depression”), occurring in approximately 49.5% of Bipolar II patients (Benazzi, 2004a). According to the bipolar spectrum notion, these symptoms are common, dimensional and frequently recurring (Perugi et al., 2001; Benazzi, 2004b; Moreno and Andrade, 2005). Their presence can significantly affect the course of the disease (Goldberg et al., 2009), with a growing tendency toward the destabilization of mood (Goldberg et al., 2007), recurrent syndromes (Perlis et al., 2006) and an increased rate of suicidality (Goldberg et al., 1998). Recent evidence suggests that mixed states, even in their sub-threshold forms, can influence the course and outcome of bipolar depression in the medium term, compared to the “pure” form of the disease (Dodd et al., 2010; Mazza et al., 2011) and there is a substantial impact on overall functioning, social relationships and perceived well-being (Mazza et al., 2011). It seems that mixed states may be underestimated by both clinicians and patients, leading to a decrease of the rate of recognition and the possibility of establishing a more specific treatment (Mazza et al., 2012).

Psychometric scales normally used for the evaluation of depressive symptoms, such as Hamilton Depression Rating Scale-HDRS (Hamilton, 1960) and Montgomery Asberg Depression Rating Scale-MADRS (Montgomery and Asberg, 1979), are also used to assess BD patients, but lack in distinguishing bipolar depression, expecially with mixed and atypical symptoms (Hantouche and Akiskal, 2005; Serretti and Olgiati, 2005).

The Bipolar Depression Rating Scale (BDRS) is a purpose-built instrument (Berk et al., 2007) designed to capture the unique clinical characteristics of bipolar depression. It is a useful tool not only for research but also in daily practice. The scale consists of 20 items, each with a score from 0 to 3 for a maximum total of 60 points. A semi-structured interview has been built together with the instrument, in order to limit the variability between test administrators using standardized anchor-points.

The aim of our study is the validation and the reliability of BDRS in an Italian sample of bipolar patients. We built an inter-university collaborative group (Catholic University of Scared Heart of Rome; University of Bari; University of Florence; University of Bologna; Libera Università degli Studi Maria SS. Assunta-LUMSA of Rome) in order to decrease the chance of validation biases, such as heterogeneity of patients, different recruitment possibilities (outpatients and inpatients) and the variability of symptom manifestations, concordant with significant socioeconomic differences between North and South of Italy (Pompili et al., 2008).

The main purpose of the present study was the validation of BDRS among an Italian population for use both for research and in everyday clinical activity. The secondary aim was the evaluation, among separate clinical samples, of differences between unipolar and bipolar depression and between bipolar I and II depression. Finally, we investigated temperamental and character features in different samples of depressed patients with Cloninger's TCI (Martinotti et al., 2008), in order to find any common personological trait of bipolar depression as previously inspected by some authors (Cloninger et al., 1998; Harley et al., 2011)

## Materials and methods

The study was conducted in accordance with the latest revision of the Declaration of Helsinki and the rules of Good Clinical Practice (ICH-GCP). The Institutional Ethical and Review Board of the Catholic University of Sacred Heart in Rome approved the study with protocol (P/521/CE/2011). All subjects provided written informed consent after a complete description of the study procedures and participated without receiving any form of payment.

### The I-BDRS

In order to achieve comparability between the Italian translation of BDRS and the original instrument, we used a multiple-phase translation process based on the 5 cross-cultural criteria of Flaherty (Flaherty et al., 1998) and general guidelines for translating study instruments. Firstly two independent teams (Rome and Bari) translated the original scale into Italian; then the translations were compared and integrated into a single version. The next step was a back translation into English by a native speaker, followed by a consistency check by the original authors.

The Italian version of the BDRS, as the original instrument, consists of 20 items each with a score ranging from 0 to 3, with a minimum of 0 and a maximum score of 60.

### Study sample

Subjects were recruited from July 2011 to July 2015 among outpatients referred to the Bipolar Disorder Unit of the A. Gemelli Hospital in Rome and among inpatients and outpatients admitted to the Psychiatric Ward of the Hospital Policlinico Consorziale in Bari.

Inclusion criteria were: (Judd et al., 2002) currently meeting DSM-IV-TR criteria for Bipolar Disorder I (BP-I), Bipolar Disorder II (BP-II) (2) age 18–75 years; (3) current experience of depressive symptoms (but not necessarily fulfilling criteria for a major depressive episode); (4) native Italian speakers, with mastery of spoken and written Italian language.

Subject were excluded if any of the following conditions were present: (1) a diagnosis of mental retardation or documented IQ < 70; (2) any other DSM diagnosis (3) unstable general medical conditions; (3) clinically significant pre-study physical exam, electrocardiogram, laboratory or urinalysis abnormalities indicating serious medical disease impairing evaluation; (4) pregnant or breast-feeding women. A sample of patients with a current diagnosis of Major Depressive Disorder (MDD), fulfilling the same inclusion/exclusion criteria was recruited.

At evaluation, patients followed a naturalistic maintenance treatment, with typical or atypical antipsychotics (Asenapine, Aripiprazole, Clotiapine, Clozapine, Haloperidole, Olanzapine, Quetiapine, Risperidone, and Paliperidone), mood stabilizers or antiepileptic drugs (Lithium, Valproate, Carbamazepine, Lamotrigine, and Oxcarbazepine), antidepressants (SSRI, SNRI, NaSSA, and other unspecific antidepressants) and Benzodiazepines or Hypnotics.

### Procedure

A BP diagnosis was established by trained psychiatrists using the Structured Clinical Interview for DSM-IV Axis I Disorder (SCID-I) (First et al., 1996). Personality disorders were excluded through the Structured Clinical Interview for DSM-IV Axis II Disorders (SCID-II) (First et al., 1997). An anamnestic interview was administered in order to obtain sociodemographic information and psychiatric history.

All participants were interviewed by specifically trained psychiatrists using the Bipolar Depression Rating Scale (BDRS), HDRS 21-item version (HAM-D), Montgomery Asberg Depression Rating Scale (MADRS) and Young Mania Rating Scale (YMRS). Good inter-rate reliability has been found on all instruments (Fleiss' coefficient 0.82). Patients were further interviewed using the Italian version of the Temperament and Character Inventory revised version (TCI-R), a self-administered interview for personality characteristics (Martinotti et al., 2008).

Anonymity was guaranteed to all the participants: data were de-identified before any further data manipulation from the coordinating center of the Catholic University of the Sacred Heart. Then the database was sent to the University of Bologna, University of Florence and the LUMSA University of Rome for the statistical analysis, ensuring an adequate level of protection using a double level of data encryption.

### Statistical analysis

The distribution of the I-BDRS scores was performed, in order to test whether each item had a normal distribution, using the Kolmogorov-Smirnov one-sample test (K–S test). Descriptive statistics and Pearson's correlation tests between the different rating scales were also used. A distribution can be considered approximately normal whether skewness and kurtosis indices are between −1 and +1 (Joanes and Gill, 1998). Pearson's correlation coefficients between I-BDRS sub-scales, MADRS, HAM-D and YMRS were calculated to assess the convergent validity. In order to test discriminant validity, Student *t*-tests for independent samples were carried out between the bipolar sample and unipolar sample.

Then an exploratory factor analysis (EFA) was carried out of the I-BDRS through the principal component analysis method using IBM SPSS Statistics 21 software. To test sampling adequacy, we measured Kaiser–Meyer–Olkin (KMO) and Bartlett's test of sphericity. A sample can be considered numerically adequate when KMO is close to 1 or Bartlett's test is significant. The number of components extracted was based on the percentage of variance accounted for by the Kaiser-Guttman method and, overall, the scree plot (Mazza et al., 2012). After the selection of the number of components, we verified, by the means of the communality matrix, whether the factor model adequately represented each of the initial variables (each variable should have a score ≥0.10; a score <0.10 indicates that the variable is not properly reproduced in the factor solution). Subsequently, we verified the component loading of each variable, using the component loading matrix, in order to organize and accomunate each item with his latent factor consinstency. Then we performed a confirmatory factor analysis and carried out structural equation modeling by the use of M-PLUS. This analysis allows to test the goodness of the factor structure emerging from an exploratory model. To test the goodness of fit, we considered absolute fit indices as standardized root mean square residual (SRMR) and root mean square error adjustment (RMSEA) and incremental fit indices as comparative fit index (CFI), incremental fit index (IFI) and non-normed fit index (NNFI). Furthermore, we considered chi-square divided by degrees of freedom (χ^2^/df). As suggested by Byrne (Byrne, 1998), a model can be considered reasonably standard if SRMR and RMSEA are lower than 0.08, if CFI, IFI, and NNFI are higher than 0.90 and if χ^2^/df is <3.

The Internal consistency reliability of the scale was tested through Cronbach's alpha coefficient and, in order to explore further variables, it was calculated separately for gender and for each subtype of BD. For Cronbach's alpha interpretation, George and Mallery (George and Mallery, 2003) provided the following rules: α > 0.9 = Excellent, α > 0.8 = Good, α > 0.7 = Acceptable, α > 0.6 = Questionable, α > 0.5 = Poor, and α < 0.5 = Unacceptable.

The analysis of variance (ANOVA) was used to correlate the scores of specific sub-groups, distinguishing between BD I, BD II and unipolar patients. Finally, considering that we had more than two raters, the inter-rater reliability was measured using Fleiss' coefficient (which measures the agreement between a fixed number of raters) instead of the classic Cohen's kappa (valid only on two raters), by the independent rating of five audio-recorded interviews.

## Results

### Demographic and clinical data

On a total of 250 subjects consecutively screened, 188 patients were selected using the aforementioned criteria. Among 188 depressed patients, 63 meet criteria for MDD (33.3%), 62 for BD I (32.8%), and 63 for BD II (33.3%); 14 patients (7.4%) display some significant manic symptoms (YMRS≥12) indicating a mixed state. Bipolar patients gender ratio is 55M/69F whilst in unipolar depression there are more woman (17M/46M) and the age of onset differ from 28.3 years old of bipolar disorder vs. 38.7 years old of major depression. Other clinical characteristics of the sample are shown in Table 1, detailed for different types of depression (Bipolar and Unipolar) and sites of screening. Table 2 shows pharmacological treatment.

**Table 1:** Clinical characteristics of the sample.

**VARIABLES**	**Bipolar disorder**			**Major depressive disorder**		
	**Rome (*n* = 84)**	**Bari (*n* = 41)**	**Total sample (*n* = 125)**	**Rome (*n* = 43)**	**Bari (*n* = 20)**	**Total sample (*n* = 63)**
	***Mean ± SD***	***Mean ± SD***	***Mean ± SD***	***Mean ± SD***	***Mean ± SD***	***Mean ± SD***
Gender, Male/Female	35/48	20/21	55/69	15/28	2/18	17/46
Diagnosis, BD I/BD II	24/60	38/3	63/62			
Age (years)	50.2 ± 10.8	41.2 ± 11.3	47.2 ± 11.7	52.2 ± 12.2	52.1 ± 11.0	52.2 ± 11.8
Marital status (engaged/not engaged)	47/37	16/25	63/62	24/19	12/8	36/27
Education (years)	13.4 ± 3.4	9.4 ± 3.3	12.1 ± 3.8	13.4 ± 3.2	10.0 ± 2.9	12.9 ± 3.44
Employment (employed/not employed)	52/32	20/21	72/53	21/22	12/8	29/34
Age at onset (years)	28.9 ± 10.1	27.2 ± 9.1	28.3 ± 9.7	41.3 ± 13.5	33.3 ± 11.1	38.7 ± 13.2
Duration of illness (years)	20.8 ± 10.9	14 ± 11.3	18.3 ± 11.6	7.5 ± 4.5	14 ± 11	11.8 ± 9.7
Mood episodes (n^*^)	9.1 ± 7.7	6.9 ± 6.9	8.3 ± 7.5	3 ± 1.2	1 ± 1	1 ± 1.6
MADRS score	18.3 ± 8.3	29.5 ± 8.4	21.9 ± 9.8	23.6 ± 9.1	27.5 ± 7.1	26.2 ± 7.8
HAM-D score	17.9 ± 7.8	23.1 ± 4.6	19.6 ± 7.3	23 ± 8.70	22 ± 5.4	22.3 ± 6.4
YMRS score	5.4 ± 5.61	6.02 ± 3.27	5.6 ± 4.9	2.4 ± 1.1	4.5 ± 2.6	3.8 ± 2.4
I-BDRS score	21.1 ± 9.08	28.2 ± 5.14	23.5 ± 8.6	24.8 ± 6	28.7 ± 7.8	27.5 ± 7.4

*I-BDRS, Italian Bipolar Depression Rating Scale; HAM-D, Hamilton Depression Rating Scale; YMRS, Young Mania Rating Scale, MADRS, Montgomery Asberg Depression Rating Scale*.

* *Statistically significant*.

**Table 2:** Psychopharmacological treatments.

	**Bipolar disorder**			**Major depressive disorder**		
	**Rome (*n* = 84)**	**Bari (*n* = 41)**	**Total sample (*n* = 125)**	**Rome (*n* = 43)**	**Bari (*n* = 20)**	**Total sample (*n* = 63)**
Lithium	17	13	30	2	1	3
Mood stabilizers	66	22	88	9	0	9
Antipsychotics	44	30	74	6	5	11
Tryciclics	6	6	12	4	2	6
SSRI	18	14	32	28	11	39
MAOI	6	4	10	0	0	0
SNRI	12	6	18	6	7	13
Dopaminergic drugs	13	6	19	10	3	13
Benzodiazepines	42	20	62	5	10	15
Other drugs	17	0	17	4	0	4

### Distribution of items

**Figure 1:**
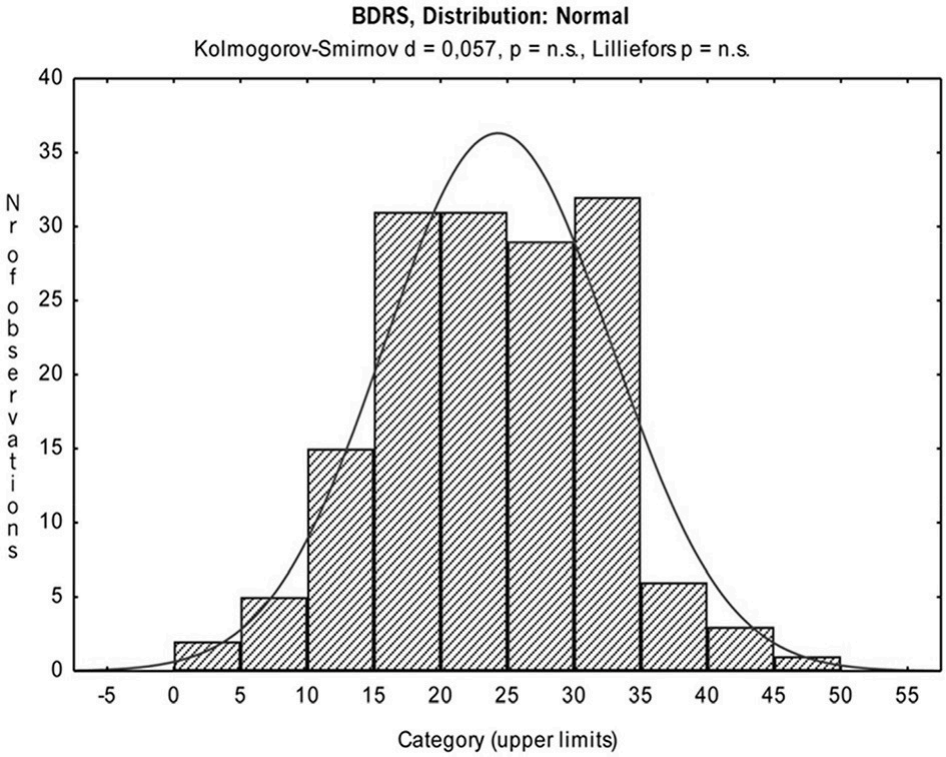
I-BDRS Scores Distribution.

Mean, standard deviation, skewness and kurtosis for each item were calculated, in order to test whether the distribution is approximately normal (see Figure 1 and Table 3). Results showed that no item had extreme means or standard deviation close to zero; furthermore, skewness and kurtosis were between −1 and +1, except that for six items for which they were slightly lower or higher. These results suggested that the item distribution could be considered approximately normal.

**Table 3:** Mean, standard deviation and kurtosis for each item of the I-BDRS.

	**Mean**	**Standard deviation**	**Skewness**	**Kurtosis**
1 - Depression	1.77	0.76	−0.46	0.12
2 - Sleep disturbance	1.23	1.04	0.17	−1.23
3 - Appetite disturbance	0.97	1.03	0.51	−1.13
4 - Social impairment	1.62	0.99	0.03	−0.87
5 - Activity/Energy	1.50	0.82	−0.01	−0.49
6 - Motivation	1.46	0.91	0.20	−0.45
7 - Concentration	1.37	0.80	0.30	−0.28
8 - Anxiety	1.92	0.81	−0.60	0.14
9 - Anhedonia	1.60	0.90	−0.20	−0.68
10 - Flattened affect	1.30	0.83	−0.01	−0.65
11 - Worthlessness	1.33	0.92	0.24	−0.74
12 - Helplessness	1.19	0.87	0.29	−0.58
13 - Suicidal ideation	0.50	0.78	1.44	1.26
14 - Guilt	1.38	0.86	0.20	−0.55
15 - Psychotic symptoms	0.62	0.82	1.18	0.56
16 - Irritability	0.72	0.81	0.93	0.23
17 - Lability	1.10	0.85	0.14	−0.95
18 - Motor drive	0.45	0.72	1.55	1.70
19 - Speech	0.46	0.67	1.13	0.07
20 - Agitation	0.96	0.76	0.41	−0.22

A summary statistic of the scales shown a mean of 24.32, a Standard Deviation of 8.46, with a variance of 71.62. The Cronbach's alpha is 0.81 and the Standardized alpha results in 0.82.

### Exploratory factor analysis

We tested the psychometric properties and the dimensional structure of the BDRS through the principal component analysis. In the preliminary analysis, KMO is equal to 0.85 and Bartlett's test is significant ($\chi_{\left(190\right)}^{2}$ = 1277.59; *p* < 0.001), suggesting our sample results are adequate. The scree plot suggests that three factors should be extracted; they have eigenvalues > 1 and explain 47.38% of the total variance. Communalities are observed between 0.19 and 0.66, suggesting that each item is well-represented by the factorial model. Table 4 shows the component loading matrix (just loads >0.30), eigenvalues and the percentages of variance accounted for each dimension.

**Table 4:** EFA—Component loading matrix, eigenvalues, and the percentages of variance accounted for each dimension.

	**Secondary depression symptoms**	**Mixed symptoms**	**Primary depression symptoms**
Motivation	0.80		
Anhedonia	0.79		
Flattened affect	0.79		
Worthlessness	0.66		0.41
Social impairment	0.65		
Activity/Energy	0.63		0.39
Guilt	0.38	0.35	
Motor drive		0.72	
Agitation		0.67	
Lability		0.66	
Speech		0.65	
Irritability		0.64	0.32
Psychotic symptoms		0.55	
Suicidal ideation			0.70
Depression	0.33		0.69
Appetite			0.62
Concentration	0.38		0.54
Helplessness	0.49		0.50
Anxiety		0.38	0.45
Sleep			0.33
Eigenvalues	5.58	2.81	1.57
Explained variance	27.88%	14.05%	7.59%

The first factor was denominated “primary depression symptoms” because it concerns aspects such as suicidal ideation, depressed mood, appetite disturbance and impaired concentration. The second factor was denominated “mixed symptoms” because it concerns aspects such as lability, increased motor drive, increased speech or agitation. Finally, third factor was denominated “secondary depression symptoms” because it concerns aspects such as reduced motivation, anhedonia, affective flattening, or worthlessness.

### Confirmatory factor analysis

**Figure 2:**
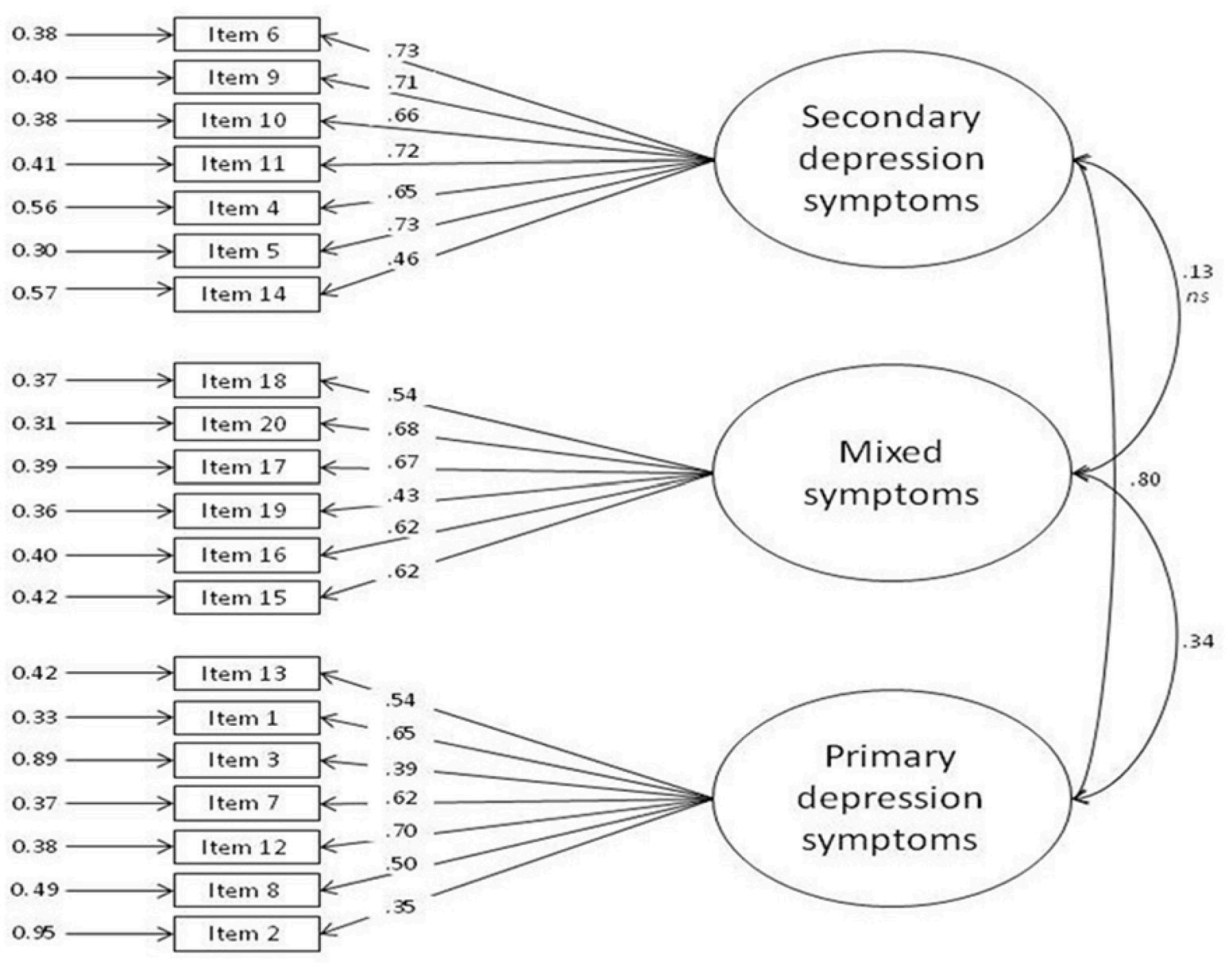
Factor Analysis.

On the basis of the results of the component analysis, the hypothetical model (Figure 2) consists of 3 latent variables (primary depression symptoms, mixed symptoms and secondary depression symptoms, represented in the ellipses) and 20 observed variables (the items, represented in a box).

The model reached the following fit indices: SRMR = 0.080, RMSEA = 0.071, CFI = 0.861, and NNFI = 0.853; furthermore, the χ^2^/df was 1.68 (279.77/167). Therefore, the results suggested that the model fits the data reasonably well, although CFI an d NNFI were slightly lower than 0.90.

### Correlation between rating scales

The Italian BDRS total has strong positive correlation coefficients with HAM-D (*r* = 0.81, *p* < 0.001) and with MADRS (*r* = 0.67, *p* < 0.001) and, in contrast to the original validation sample of Berk et al. (2007), a good correlation with YMRS (*r* = 0.46, *p* < 0.001). The analysis of variance (ANOVA) between total scores in BD I, BD II, and MDD patients is shown in Table 5.

**Table 5:** ANOVA Total Scores.

	**MADRS**		**HAM-D**		**YMRS**		**I-BDRS**	
	**Mean**	***SD***	**Mean**	***SD***	**Mean**	***SD***	**Mean**	***SD***
BD I (*n* = 62)	26.5	10.3	22.7	7.3	7.4	5.6	27.1	8.2
BD II (*n* = 63)	17.6	7.2	16.7	6.2	3.8	3.5	20.0	7.7
MDD (*n* = 63)	51.7	161	23.9	7.2	4.5	3.5	27.5	7.1
*F, p*	105.5	<0.0001	16.8	<0.0001	16.0	<0.0001	11.2	<0.0001

*BD, Bipolar Disorder; I-BDRS, Italian Bipolar Depression Rating Scale; HAM-D, Hamilton Depression Rating Scale; YMRS, Young Mania Rating Scale, MADRS, Montgomery Asberg Depression Rating Scale; MDD, Major Depressive Disorder*.

### Reliability of the scale

The internal consistency reliability of the scale, assessed by calculation of Cronbach's alpha, as mentioned shows that I-BDRS has a score of 0.82. This good result is coupled with the excellent values obtained on the total index of skewness and kurtosis, both below zero with a decline of tails better than average. The Kolmogorov-Smirnov test gave a similar result (*d* = 0.057; *p* = 0.92), confirming a normal distribution (see Figure 1).

In order to explore the possible effect of gender or the impact of sub-diagnosis on internal consistency of the instrument, the Cronbach's alpha of the different subgroups is shown in Table 6.

**Table 6:** Reliability of the scale.

	**BD males**	**BD females**	**BD I**	**BD II**	**MDD**	**BD Total**
Mean	24.19 ± 7.99	24.33 ± 8.80	27.08 ± 8.16	20.09 ± 7.59	27.62 ± 7.09	24.32 ± 8.46
Variance	63.77	77.41	66.60	57.64	50.32	71.62
Skewness	−0.70	−0.27	−0.40	−0.32	−0.06	−0.05
Kurtosis	−0.64	−0.62	−0.71	−0.02	−0.15	−0.25
Minimum	2	8	5	2	12	2
Maximum	42	46	46	40	41	46
Cronbach's α (raw)	0.79	0.83	0.77	0.85	0.77	0.81
Inter-item correlation	0.17	0.21	0.15	0.25	0.16	0.19
Cronbach's α (stand.)	0.80	0.83	0.77	0.86	0.78	0.82

*BD, Bipolar Disorder; MDD, Major Depressive Disorder*.

Pearson's correlation coefficient between each item and the total BDRS score (item total correlations) is shown in Table 7: all items significantly correlated with the total score at the 0.05 level of significance. For a better understanding of the instrument's reliability and sensibility, the same analysis was performed for the 3 sub-samples (BD I, BD II, and MDD patients), results are shown in Table 7.

**Table 7:** Reliability on different sub-samples.

	**TOTAL**				**MDD**				**BD 1**				**BD 2**			
	***M***	***SD***	**I-T**	**α**	***M***	***SD***	**I-T**	**α**	***M***	***SD***	**I-T**	**α**	***M***	***SD***	**I-T**	**α**
Depression	1.77	0.76	0.21	0.81	0.62	1.08	0.04	0.78	1.90	0.76	0.34	0.76	1.63	0.75	0.62	0.83
Sleep	1.23	1.04	0.23	0.81	0.21	0.49	0.45	0.75	1.58	1.03	0.12	0.78	0.89	0.94	0.43	0.84
Appetite	0.97	1.03	0.34	0.81	2.28	0.53	0.50	0.75	1.24	1.05	0.22	0.77	0.70	0.94	0.23	0.85
Social impair.	1.62	0.99	0.54	0.80	1.41	0.95	0.45	0.75	1.84	1.09	0.56	0.75	1.43	0.82	0.60	0.83
Activity/Energy	1.50	0.82	0.46	0.80	1.34	1.11	0.35	0.76	1.71	0.86	0.42	0.76	1.30	0.73	0.57	0.83
Motivation	1.46	0.91	0.49	0.80	1.83	0.93	0.45	0.75	1.52	1.05	0.40	0.76	1.40	0.75	0.67	0.83
Concentration	1.37	0.80	0.54	0.80	1.86	0.58	0.58	0.75	1.61	0.93	0.50	0.75	1.13	0.55	0.40	0.84
Anxiety	1.92	0.81	0.18	0.82	1.69	0.66	0.10	0.77	1.98	0.91	0.49	0.75	2.02	1.34	0.10	0.87
Anhedonia	1.60	0.90	0.49	0.80	1.86	0.79	0.33	0.76	1.68	1.00	0.51	0.75	1.52	0.78	0.54	0.83
Flattened aff.	1.30	0.83	0.46	0.80	2.10	0.82	0.39	0.75	1.34	0.90	0.30	0.77	1.27	0.75	0.73	0.83
Worthlessness	1.33	0.92	0.53	0.80	1.62	0.90	0.27	0.76	1.50	1.11	0.51	0.75	1.16	0.65	0.65	0.83
Helplessness	1.19	0.87	0.56	0.80	1.41	0.73	0.43	0.75	1.42	0.97	0.54	0.75	1.02	0.73	0.55	0.83
Suicidal ideat.	0.50	0.78	0.40	0.80	1.86	0.88	0.42	0.75	0.65	0.89	0.38	0.76	0.37	0.63	0.36	0.84
Guilt	1.38	0.86	0.50	0.80	1.83	0.80	0.49	0.75	1.61	0.95	0.43	0.76	1.14	0.69	0.42	0.84
Psychotic sym.	0.62	0.82	0.38	0.80	0.86	0.95	0.50	0.74	0.97	0.94	0.19	0.77	0.27	0.48	0.30	0.84
Irritability	0.72	0.81	0.35	0.81	1.62	0.62	0.47	0.75	1.02	0.90	0.18	0.77	0.43	0.59	0.20	0.85
Lability	1.10	0.85	0.26	0.81	0.66	1.04	0.29	0.76	1.32	0.92	0.13	0.78	0.87	0.71	0.42	0.84
Motor drive	0.45	0.72	0.29	0.81	1.03	0.87	0.31	0.76	0.61	0.84	0.03	0.78	0.29	0.55	0.40	0.84
Speech	0.46	0.67	0.21	0.81	1.17	0.80	0.01	0.78	0.60	0.76	0.04	0.78	0.33	0.54	0.28	0.85
Agitation	0.96	0.76	0.26	0.81	0.34	0.61	0.07	0.77	0.98	0.86	0.33	0.76	0.94	0.64	0.57	0.83

*BD, Bipolar Disorder; MDD, Major Depressive Disorder; SD, Standard Deviation; I-T, Item Total*.

### Reliabilty, convergent and discriminant validity

Table 8 reports the Cronbach's alpha for each scale of the BDRS and the Pearson correlations between the BDRS's subscales and other measures.

**Table 8:** Cronbach's Alpha and the Pearson correlations between the I-BDRS's subscales and the other measures.

	**Secondary depression symptoms**	**Mixed symptoms**	**Primary depression symptoms**
MADRS	0.70^**^	0.29^**^	0.85^**^
HAM-D	0.72^**^	0.41^**^	0.70^**^
YMRS	0.21^*^	0.74^**^	0.20^*^
	α = 0.84	α = 0.75	α = 0.75

*HAM-D, Hamilton Depression Rating Scale; YMRS, Young Mania Rating Scale; MADRS, Montgomery Asberg Depression Rating Scale*.

*, ** *Statistically significant*.

The I-BDRS's subscales had good internal consistency coefficients and therefore they can be considered reliable, as suggested by Cronbach's alpha coefficients between 0.75 and 0.84. Furthermore, the measures concerning depression (MADRS and HAM-D) were positively related to the I-BDRS's subscales, but mostly to the two subscales measuring depression (primary and secondary). Similarly, the measure concerning mania was positively related to I-BDRS's subscales, but mostly to subscale measuring mixed symptoms. Moreover I-BDRS scores, controlling for age and sex, were significantly higher in BD1 (*F* = 7.34 *p* = 0.001). Higher scores in BD I were observed also on MADRS scores (*p* < 0.001) and HAMD (*p* = 0.001). The BD I group also had higher scores on the YMRS (*p* < 0.001).

We then tested differences on I-BDRS single items (BD I vs. BD II vs. MDD), controlling for initial severity, other than age and sex. In BD I we found significantly higher scores on the psychotic item (*p* = 0.001) and agitation item (*p* = 0.001) whilst BD II patients scored higher on flattened affect (*p* = 0.001) and anhedonia (*p* = 0.003). MDD patients showed higher scores on hopelessness (*p* = 0.002).

Finally, in order to test discriminant validity, we compared the scores of the bipolar sample with the score of unipolar sample considering the 3 subscales. In particular, we hypothesized that the bipolar group would have higher scores than the unipolar group in terms of mixed symptoms, while there would not be significantly differences in primary and secondary depression. The results of *t*-tests for independent samples supported these hypotheses; in primary and secondary depression the means of the bipolar group (*M* = 1.28 and *sd* = 0.54; *M* = 1.45 and *sd* = 0.64, respectively) were similar to the unipolar group (*M* = 1.41 and *sd* = 0.57; *M* = 1.48 and *sd* = 0.61, respectively).

In mixed symptoms, the mean of the bipolars group (*M* = 0.72 and *sd* = 0.52) was significantly higher (*p* < 0.05) than the unipolar group (*M* = 0.56 and *sd* = 0.45), suggesting that the BDRS was able to distinguish between unipolar and bipolar depressed patients. These positive correlations supported the convergent and discriminant validity of the I-BDRS.

### TCI-R and BDRS correlation

The correlation between the total score of BDRS and TCI-R subscales is shown in Table 9. No evident correlation was found in any of the TCI-R subscales.

**Table 9:** I-BDRS and TCI-R correlations.

	***R/p***		***R/p***		***R/p***		***R/p***
NS1	0.0019	HA	0.1300	PS4	−0.0232	C2	−0.1046
	*p* = 0.987		*p* = 0.280		*p* = 0.847		*p* = 0.385
NS2	0.0910	RD1	−0.0572	PE	0.0012	C3	−0.1403
	*p* = 0.450		*p* = 0.635		*p* = 0.992		*p* = 0.243
NS3	0.0660	RD2	−0.1340	SD1	−0.0781	C4	−0.0768
	*p* = 0.585		*p* = 0.265		*p* = 0.517		*p* = 0.524
NS4	−0.0320	RD3	0.0727	SD2	−0.2425	C5	−0.0802
	*p* = 0.791		*p* = 0.547		*p* = 0.042		*p* = 0.506
NS	0.0417	RD4	0.0419	SD3	−0.1511	C	−0.1184
	*p* = 0.730		*p* = 0.729		*p* = 0.208		*p* = 0.325
HA1	0.2145	RD	0.1945	SD4	−0.1235	ST1	0.0278
	*p* = 0.072		*p* = 0.104		*p* = 0.305		*p* = 0.818
HA2	0.0404	PS1	0.0308	SD5	−0.1366	ST2	−0.0495
	*p* = 0.738		*p* = 0.799		*p* = 0.256		*p* = 0.682
HA3	0.0679	PS2	−0.0692	SD	−0.1539	ST3	−0.0123
	*p* = 0.574		*p* = 0.567		*p* = 0.200		*p* = 0.919
HA4	0.0952	PS3	0.0617	C1	−0.1209	ST	−0.0098
	*p* = 0.430		*p* = 0.609		*p* = 0.315		*p* = 0.935

*NS, Novelty Seeking HA; Harm Avoidance; RD, Reward Dependance; PE, Persistance; SD, Self Directedness; C, Coperativeness; ST, Self Trascendence*.

## Discussion

To the best of our knowledge this is the first published psychometric study of a scale for the assessment of bipolar depressive symptoms on an Italian sample.

### Validity and reliability of the scale

The results show that the I-BDRS is a valid scale for the measurement of depression in patients with Bipolar Disorder, with a considerable internal consistency (Cronbach's α 0.82), a significant item-total correlations (although in a range from 0.18 to 0.56) and strong positive correlation with the depressive symptom severity measured by the other administered scales (MADRS *r* = 0.67, *p* < 0.001; HAM-D *r* = 0.81, *p* < 0.001; YMRS *r* = 0.46 *p* < 0.0001), including the YMRS (different to other samples in the literature and to the original validation sample) (Berk et al., 2007). There is a good inter-rater reliability, measured by Fleiss' coefficient with a mean result of 0.81. The Cronbach's alpha remains high even in the different subsamples, confirming that there is no impact of gender as well as Bipolar I or II diagnosis. These robust results are noteworthy, expecially when considering the notable representativeness of demographic distributions of the sample that includes bipolar inpatients and outpatients, tested in two separated Italian centres, with quite different catchment areas. The clinical characteristics of the sample had favorable elements including an even BP I/BP II ratio (62/63) and expected differences between unipolar and bipolar patients (higher number of females in MDD, more episodes and a longer illness in BD), confirming patterns seen in other samples reported in literature (Weissman et al., 1996; Kessler et al., 2003; Berk et al., 2007; Mazza et al., 2011, 2012; Nivoli et al., 2011).

### The I-BDRS structure

We showed that a three-cluster structure of the instrument is the most parsimonious model, in line with the original construction of the scale that included two sections for depression and one for mixed symptoms.

As with other scales such as the Beck Depression Inventory (BDI) and other international validations of the BDRS (Galvão et al., 2013; Sarró et al., 2015) the factors related to a psychological component of depression (Anhedonia, Reduced Motivation, Flattened Affect, Worthlessness, Social Impairment, Reduced Energy) are associated in a specific sub-scale, and the impact of the core component of depression (Mood, Suicidal Ideation, Change in Appetite and Sleep) is expressed by another sub-scale. Other items typical of mixed states (Motor Drive, Agitation, Lability, increased speech, Irritability and Psychotic Symptoms) were carried in the same cluster, constituting the subscale for mixed depression.

### Differences between BD and MDD

The patterns of item's mean scores give an indication of how the symptoms perform in terms of salience for this sample. The highest mean load fell between the mild and moderate anchor point. The most highly expressed items were anxiety, anedonia, social impairment, depressed mood and reduced activity and the least loaded items were irritability, increased motor drive, increased speech, and agitation. This is compatible with literature suggesting that mixed states are present in a significant minority of individuals with bipolar depression (Benazzi, 2004a).

MDD patients showed higher mean in MADRS and HAM-D total scores but similar scores on the BDRS compared to Bipolar Disorders. A possible explanation could be addressed to the structure of the scales, expecially if we take into account the results of the item-total correlation of MDD patients in BDRS: the main factor loading for the total score is the summary of classical depressive symptoms (like reduced concentration, helplessness, guilt, social impairment and reduced motivation) and physical symptoms (linked biological and circadian rhythms such as appetite and sleep disturbances). The patterns were completely different among the BD sample, where the factor loadings exclude appetite and sleep and are more weighted to mental symptoms and mixed symptoms of depression.

It is interesting that, in MDD patients, anxious, and pure depressive symptoms are the lowest contributors to the total: this could explain the higher scores on the MADRS vs. HAM-D (considering that MADRS items are more focused on mood symptoms while HAM-D has more items for evaluation of anxious symptoms). Regarding the ANOVA symptoms analysis between BD I and BD II, BD I patients are different in terms of psychotic symptoms and agitation, while Bipolar II patients more frequently express anhedonia and flattened affect. MDD patients are characterized by more hopelessness than BP patients. Another interesting finding is that unipolar depressed patients showed a lower load of anxious and pure depressive symptoms on the total scores of the BDRS, in agreement with other studies (Katz et al., 1982).

Our study confirmed that anhedonia could have a role in discriminating between MDD and BD depressed patients, as reported by several authors (Endicott et al., 1985; Coryell et al., 1989; Parker et al., 2000). This observation could be a possible explanation of the fact that, even if the I-BDRS scores were similar between unipolar depressed patients and Bipolar I depressed patients, MADRS and HAM-D scores differed between the two samples. Moreover, Bipolar I patients showed higher scores on the different psychometric scales, with similar means compared to those of unipolar depressed patients (except for MADRS scores that were higher in unipolar depressed patients with a higher standard deviation).

As for BDRS item correlation in the different subsamples it is noted that Bipolar I patients had few factors with a high loading (social impairment, reduced concentration, worthlessness, anxiety, helplessness, and guilt) whilst Bipolar II patients showed a more varied picture with a widespread factor loading that excludes, as in unipolar depressed patients, only anxious symptoms. From this point of view, it seems that Bipolar II depression, excluding mixed symptoms, is more similar to unipolar depression while Bipolar I depression is characterized by fewer but more expressed symptoms. Besides, patients with Bipolar I Depression patients seem to present more anxiety. It is not clear if depression with psychomotor activation and anxiety can be ascribed purely to bipolar disorder, but mixed depression can be represented as a symptomatic continuum between unipolar depression and mania, with variable expressions of bipolarity representing dimensions of underlying pathophysiologic processes (Benazzi, 2007).

### Differential diagnosys between bipolar and unipolar depression

As reported in literature (Galvão et al., 2013; Hirschfeld, 2014) psychometric instruments that can differentiate mixed symptoms could be very useful in the diffential diagnosis of Bipolar or Unipolar Depression. A Portuguese version of the BDRS (BDRS-P) already demonstrated validity in screening bipolar patients (Galvão et al., 2013). Our study contributes to outline the clinical usefulness of this instrument and confirms that BD patients score higher on the mixed symptoms subscale.

### Limits of the study

Although the sample is balanced and thus could be representative of the different characteristics of the depressed population, we have not found noticeable associations between the BDRS total scores and the subscales of the TCI-R.

The limited statistical power may have not allowed us to detect temperamental and characterial nuances of the patients. We did however exclude personality disorder, which may have weakened the capacity to find such factors. The main limitation is sample size, which reduces the strength of our findings. Another limitation could be that the assessors were not blind about the clinical history of each patient. Furthermore, the naturalistic characterization of the sample might have introduced a number of confounding factors (e.g., treatment options), though we systematically controlled for some of them.

## Conclusions

One of the major unfulfilled objectives of psychopathology is the differentiation of the clinical features of depression in major depressive disorder and bipolar disorder. A large body of evidence supports specific qualitative differences in phenomenology between unipolar and bipolar depression: some features such as lability and psychomotor retardation may suggest latent bipolarity (Perugi et al., 2001) and, in addition, bipolar depression can be characterized by subsyndromal manic or hypomanic symptoms (Mazza et al., 2011). The challenge of the diagnosis remains, considering the higher prevalence of depressive than hypomanic or manic symptoms in bipolar disorder, and high rates of subthreshold mixed symptoms in people diagnosed with unipolar depression. Based on these phenomenological differences we found that the I-BDRS could be a sensitive tool, both in pure depression and in mixed states, and could be used in the everyday screening and treatment of Bipolar Disorder.

## Author contributions

All authors conceived and designed the multicenter study; All authors collected data; AB and MM performed interpretation of data and wrote first draft of manuscript; LM and AC did all statistical analysis; All authors provided important intellectual content revising the manuscript.

### Conflict of interest statement

The authors declare that the research was conducted in the absence of any commercial or financial relationships that could be construed as a potential conflict of interest.
